# Oncometabolic surgery in gastric cancer patients with type 2 diabetes

**DOI:** 10.1038/s41598-022-15404-2

**Published:** 2022-07-13

**Authors:** Yun Suk Choi, Jin Wook Yi, Woo Young Shin, Yoonseok Heo

**Affiliations:** Department of Surgery, Inha University Hospital & College of Medicine, 27, Inhang-ro, Jung-gu, Inchon, 22332 Republic of Korea

**Keywords:** Gastroenterology, Gastrointestinal diseases, Gastrointestinal cancer, Gastric cancer

## Abstract

The rates of early gastric cancer and type 2 diabetes mellitus(T2DM) are sharply increasing in Korea. Oncometabolic surgery in which metabolic surgery is conducted along with cancer surgery is a method used to treat gastric cancer and T2DM in one-stage operation. From 2011 to 2019, a total of 48 patients underwent long-limb Roux-en-Y gastrectomy (LRYG) in Inha University Hospital, and all data were reviewed retrospectively. A 75 g oral glucose tolerance test and serum insulin level test were performed before and 1 week and 1 year after surgery. One year after LRYG operation, 25 of 48 patients showed complete or partial remission and 23 patients showed non-remission of T2DM. The preoperative HbA1c level was significantly lower and the change in HbA1c was significantly greater in the T2DM remission group. Insulin secretion indices(insulinogenic index and disposition index) were increased significantly in the T2DM remission group. In contrast, the insulin resistance indices (homeostatic model assessment of insulin resistance (HOMA-IR) and Matsuda index) changed minimal. In the case of LRYG in T2DM patients, remnant β cell function is an important predictor of favorable glycemic control.

## Introduction

Bariatric surgery as a treatment for severely obese patients is known as a very effective tool and has been widely implemented. Obese patients usually have comorbidities such as type 2 diabetes mellitus (T2DM), sleep apnea and hyperlipidemia. The concept of metabolic surgery has emerged, as T2DM improvements have been observed after bariatric surgery. Roux-en-Y gastric bypass(RYGP) is a type of bariatric surgery, and the diabetes mellitus (DM) remission rate among RYGP patients is reported to be approximately 40.6–88%^1–5^.

Approximately 60% of diabetes cases worldwide occur in Asia, the rate of increase in incidence is higher in Asia than in the West, and diabetes occurs more frequently in young people^6,7^. According to report on the Korea National Health and Nutritional Examination survey(KNHANES) 2011, 12.4% of Koreans over the age of 30 have diabetes, which is reported to be about 4 million or more^8,9^. This rapid increase in the number of diabetic patients leads to complications and mortality associated with diabetes, which induces an enormous economic burden^10^. In 2014, diabetes was the sixth leading cause of death in Korean about 3.9% of all deaths among aged 20–79 years people^11^. And diabetes is the most common cause of renal replacement therapy^12^.

With the National Cancer Screening System in Korea, the detection rate of early gastric cancer (EGC) has increased by 1.7 times compare to non-screening period and more than 50% of patients are found to have EGC^13^. As a result of early detection along with an increase in survival time and a 21% decrease in mortality rate, the 5 year survival rate of EGC has been reported to be over 90% in recent decades^14^. Therefore, surgery that can improve the quality of life of patients, such as function-preserving surgery or oncometabolic surgery, has been considered beyond surgery used only as a treatment for gastric cancer.

There were several reports that Roux-en-Y reconstruction has a better effect than other reconstruction to improve diabetes after surgery^15,16^. The reason for these results is assumed to be hormonal change according to the foregut theory and hindgut theory^17–21^. Several studies have reported hormonal changes in various lengths of biliopancreatic limbs^22,23^. In order to maximize the effect of this hormonal change, long limb Roux-en-Y gastrectomy was performed to improve T2DM, and positive results were reported in several studies^24–26^.

The rate of T2DM improvement after gastrectomy was reported to be approximately 30.4–55.7% in several previous studies; this rate is lower than that observed after bariatric surgery^15,27–30^. Few studies have evaluated factors that are important for improving T2DM after gastrectomy^15,28–30^. We will evaluate the difference between patients whose T2DM improved and those whose T2DM was unimproved after long-limb Roux-en-Y gastrectomy (LRYG) and investigate potential factors predicting T2DM improvement after oncometabolic gastrectomy.

## Patients and methods

A retrospective study was conducted with 48 gastric cancer patients with T2DM who underwent LRYG at Inha University Hospital from January 2011 to December 2019. Of these 48 patients, 36 underwent subtotal gastrectomy, and 12 underwent total gastrectomy. Among these patients, laboratory tests, including oral glucose tolerance tests (OGTTs) and serum insulin levels were performed before and 1 week and 1 year after surgery. Based on these results, various DM indices were calculated and reviewed. The insulinogenic index and disposition index were used to evaluate the patients' insulin secretory function, and the homeostatic model assessment of insulin resistance (HOMA-IR) and Matsuda index were used to evaluate the insulin resistance^31–33^. The baseline and postoperative follow-up data, glycemic control status outcomes, antidiabetic medication use and success of T2DM remission were reviewed.

### Analytic method

T2DM patients were defined as those with fasting blood sugar (FBS) > 126 mg/dL and HbA1c > 7% or those with a previous antidiabetic medication history. T2DM complete remission was defined as HbA1c < 6% and FBS < 100 mg/dL, and partial remission was defined as HbA1c < 6.5% and FBS 100–125 mg/dL without the use of antidiabetic medication 1 year after surgery. The non-remission group was defined as those who used antidiabetic medication or those whose glucose profile was not controlled to at least the partial remission level.

### Surgical method

Oncometabolic surgery was performed only on patients expected to have stage I or II on preoperative examination, because these patients are expected long term survival and no need of chemotherpy. Radical D1 + or D2 subtotal gastrectomy or total gastrectomy was performed according to the patient's gastric cancer location and stage. Conventional Roux-en- Y anastomosis is usually performed with a 20 cm jejunum limb and a 40 cm Roux limb. In contrast, for LRYG anastomosis, the jejunum limb was made 80 cm below the Treiz ligament for foregut bypassing, and a 80 cm Roux limb was made to perform anastomosis with the remnant stomach to reach the distal ileum early.

### Statistical analysis

Statistical analysis was performed using SPSS v 22.0 (SPSS Inc., Chicago, IL). The chi-square test was used to analyze categorical variables, and Student’s t-test was used to analyze continuous variables in the T2DM and non-T2DM groups. Fisher’s exact test was used to analyze categorical variables, and the Mann–Whitney test was used to analyze continuous variables in the subtotal gastrectomy subgroup analysis. Data are presented as the means with standard deviations or medians with ranges.

### Ethics

All patients were gastric cancer patients and they must perform gastrectomy. Oncometabolic surgery and OGTT were explained to all patients. Only patients who provided informed consent to oncometabolic surgery and OGTT were included and patients who did not want were excluded.

The study protocol was approved by the Institutional Review Board of Inha University Hospital (IRB number: INH 2021-01-013). All methods were performed in accordance with the relevant guidelines and regulations.

## Results

Forty patients were using oral antidiabetic drugs; among them, 5 patients were using insulin injections as well. Eight patients did not use antidiabetic treatment. 6 patients were diagnosed with T2DM for the first time before surgery and 2 patients did not use voluntarily anti diabetic medication. The T2DM remission group included 25 patients (25/45, 55.6%), and the non-remission group included 23 patients. In the T2DM remission group, 21 patients (21/45, 46.7%) achieved complete remission, and 4 patients (4/45, 8.9%) achieved partial remission. In the non-remission group, 19 patients used oral antidiabetic drugs, 1 patient used insulin injection along with oral antidiabetic drugs, and 3 patients did not use antidiabetic drugs in spite of their HbA1c > 7.0% at 1 year although they have HbA1C level < 7.0% in laboratory at 6 month, because they refuse antidiabetic drugs.

**Figure 1:**
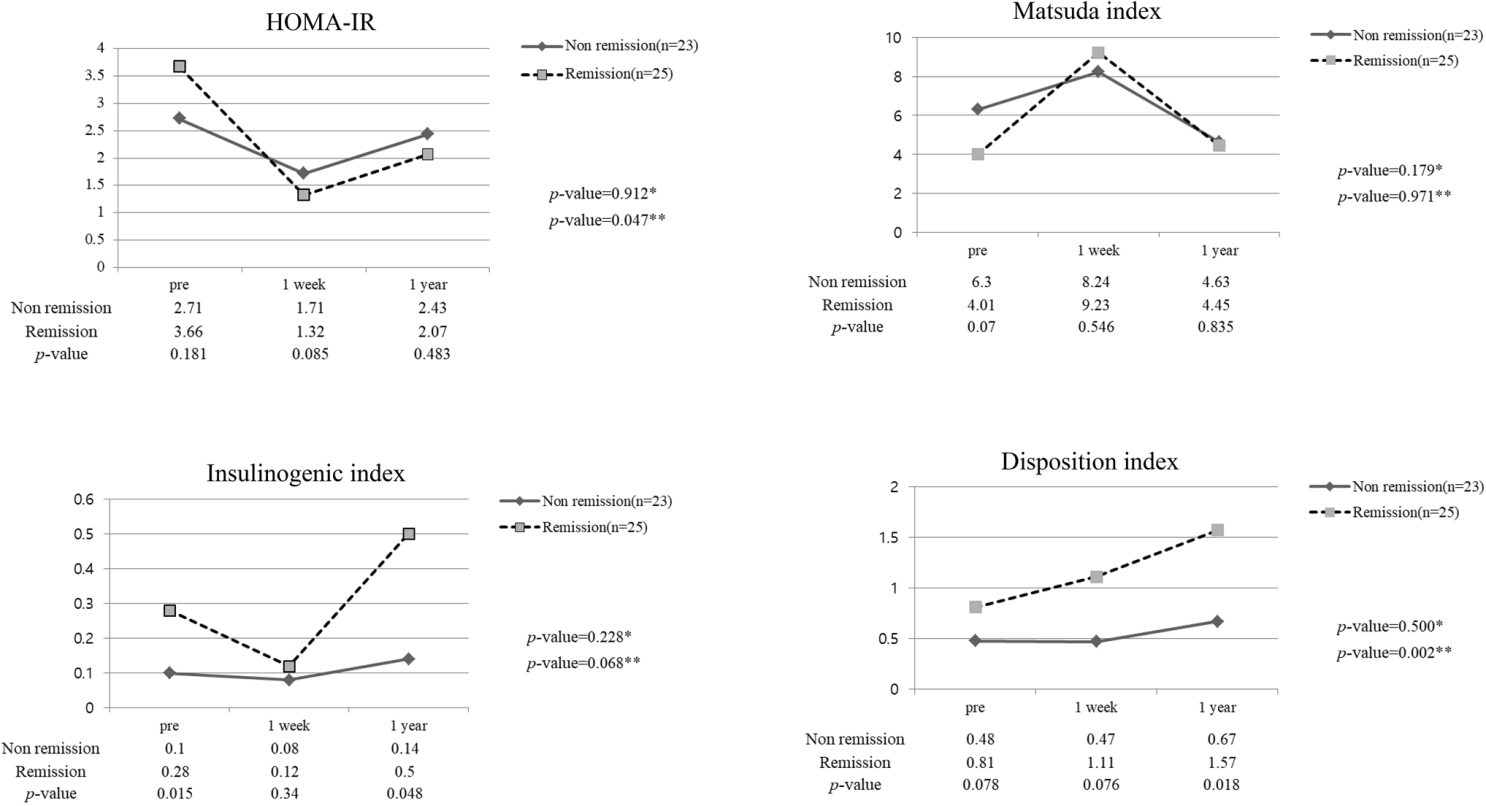
Diabetic index in long-limb Roux-en-Y gastrectomy patients (n = 48). *P value: preoperative versus 1-year postoperative value in the non-remission group. **P value: preoperative versus 1-year postoperative value in the remission group.

Table 1 shows the clinical characteristics of these two groups. Preoperative HbA1c was significantly lower in the T2DM remission group (6.95 ± 1.06% vs. 7.95 ± 1.67%; *p* = 0.002). There was a more significant difference in HbA1c levels at 1 year after LRYG (5.75 ± 0.32% vs. 7.33 ± 1.04%; *p* = 0.001). T2DM duration was shorter in remission group, but it was not statistically significant. The preoperative insulinogenic index and disposition index, which represent insulin secretory function, were significantly higher in the T2DM remission group (0.28 ± 0.30 vs. 0.10 ± 0.12; *p* = 0.015, 0.81 ± 0.78 vs. 0.46 ± 0.39; *p* = 0.077). The preoperative insulin level was also significantly higher in the T2DM remission group (12.13 ± 7.78 vs. 8.44 ± 3.31; *p* = 0.041). After 1 year, serum insulin increased more in the T2DM remission group. Insulin secretory function improved 1 year after surgery, resulting in a larger difference between the two groups than that of previous ones. In the T2DM remission group, the insulin secretory indices 1 year after surgery improved to within the normal range. However, the non-remission group did not show significant improvement (Fig. 1).

**Table 1:** Clinical characteristics of patients included in this study (non-remission vs remission).

Variable	Non-remission (n = 23)	Remission (n = 25)	*p* value
Mean age (years)	62.74 ± 9.5	58.72 ± 9.0	0.139
**Sex**			0.715
Male	17(81.0%)	23(85.2%)	
Female	4(19.0%)	4(14.8%)	
T2DM duration	8.39 ± 7.08	5.12 ± 6.13	0.093
**Preoperative diabetes medication**			
No medication	2(8.7%)	6(24.0%)	0.341
Oral medication	18(78.3%)	17(68.0%)	
Insulin injection	3(13.0%)	2(8.0%)	
BMI (kg/m^2^)-pre op BMI (kg/m^2^)-1 year	23.30 ± 3.51 22.33 ± 3.70	24.24 ± 3.14 22.64 ± 2.48	0.334 0.740
BMI change rate (%)	10.20 ± 10.06	12.38 ± 6.90	0.384
Fasting glucose (mg/dL)—pre op	123.87 ± 58.71	120.56 ± 33.66	0.814
C-peptide (ng/dL)—fast–pre op	1.79 ± 1.03	2.18 ± 1.66	0.337
C-peptide (ng/dL)—fast – 1 year	1.45 ± 0.84	1.66 ± 1.34	0.635
Insulin (mIU/L)—fast – pre op	8.44 ± 3.31	12.13 ± 7.78	0.041
HbA1C (%)—pre op	7.95 ± 1.67	6.95 ± 1.06	0.02
HbA1C (%)—1 year	7.33 ± 1.04	5.75 ± 0.32	0.001
HOMA-IR—pre op	2.71 ± 1.80	3.66 ± 2.78	0.181
Matsuda index—pre op	6.30 ± 4.65	4.01 ± 3.46	0.070
Insulinogenic index—pre op	0.10 ± 0.12	0.28 ± 0.30	0.015
Disposition index—pre op	0.48 ± 0.39	0.81 ± 0.78	0.078

*T2DM* Type 2 Diabetes Mellitus, *BMI* Body mass index, *HOMA-IR* Homeostatic model assessment of insulin resistance.

Insulin resistance indices (HOMA-IR and Matsuda index) were rather high in the T2DM remission group. In both groups, they improved rapidly 1 week after surgery and worsened again 1 year after surgery, but the patients showed improved insulin resistance status compared with that before surgery. There was no statistically significant difference between the two groups. The body mass index (BMI) change between both groups 1 year after surgery was not statistically significant.

**Figure 2:**
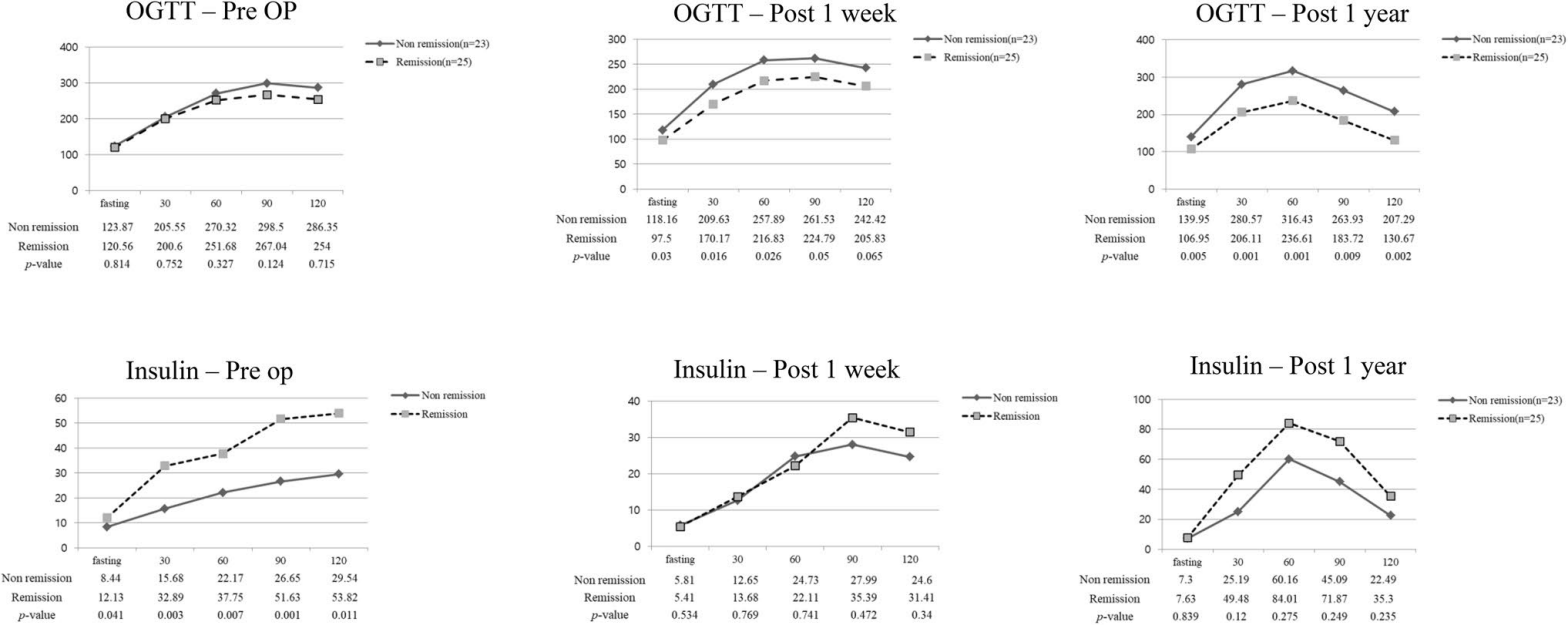
OGTT and serum insulin levels in long-limb Roux-en-Y gastrectomy patients (n = 48).

In the T2DM remission group, the OGTT results improved sharply 1 week after surgery and became almost normal after 1 year. Insulin secretion also increased to some extent in the non-remission group, and the OGTT results showed a similar pattern to that of the normal group, but the results were not in the normal range (Fig. 2).

**Figure 3:**
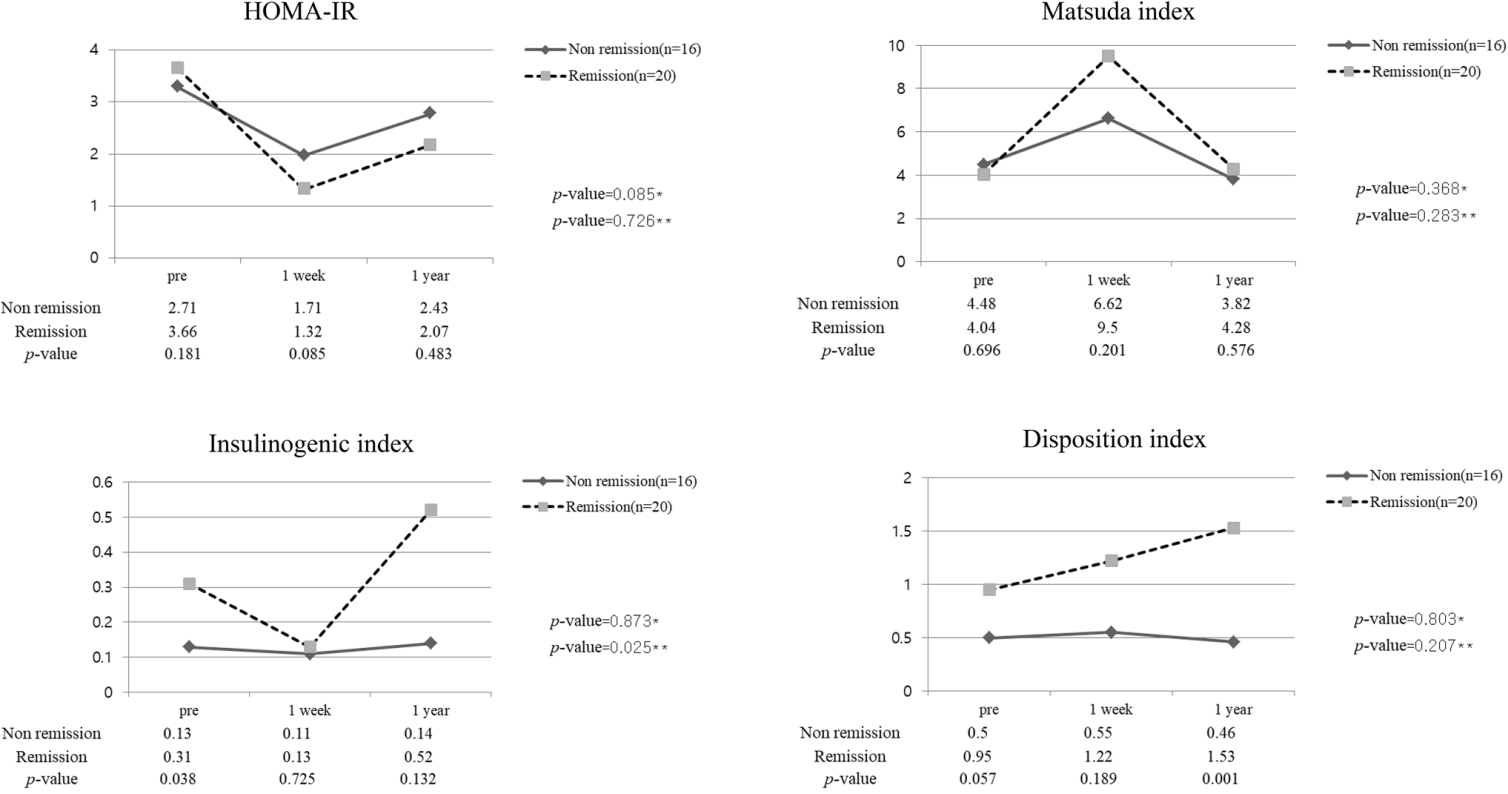
Diabetic index in subtotal long-limb Roux-en-Y gastrectomy patients (n = 36). *P value: preoperative versus 1-year postoperative value in the non-remission group. **P value: preoperative versus 1-year postoperative value in the remission group.

Among 48 patients, 36 underwent subtotal gastrectomy. Twenty patients improved and were included in the T2DM remission group, of whom 18 patients maintained their remission status, and 2 patients maintained partial remission status. Table 2 shows the clinical characteristics of the patients who underwent subtotal LRYG. Preoperative HbA1c was significantly lower in the T2DM remission group (6.76 ± 0.87% vs. 7.63 ± 1.43%; *p* = 0.045). In this group, the HbA1c level improved further, so there was a more significant difference in HbA1c after 1 year (5.75 ± 0.27% vs. 7.25 ± 1.17%; *p* = 0.002). T2DM duration was statistically significantly shorter in remission group (7.75 ± 6.48 vs. 4.10 ± 3.56; *p* = 0.039) The preoperative insulinogenic index and disposition index were significantly higher in the T2DM remission group (0.31 ± 0.32 vs. 0.13 ± 0.14; *p* = 0.038, 0.95 ± 0.83 vs. 0.50 ± 0.42; *p* = 0.057). The insulin resistance index and insulin secretory function index showed similar change patterns (Fig. 3).

**Table 2:** Clinical characteristics of patients included in the subtotal gastrectomy with long-limb Roux-en-Y bypass group (non-remission vs. remission).

Variable	Non-remission (n = 16)	Remission (n = 20)	*p* value
Mean age (years)	61.75 ± 10.33	57.85 ± 9.70	0.252
**Sex**			0.925
Male	13(81.3%)	16(80.0%)	
Female	3(18.8%)	4(20.0%)	
T2DM duration	7.75 ± 6.48	4.10 ± 3.56	0.039
**Preoperative diabetes medication**			
No medication	2(12.5%)	5(25.0%)	0.510
Oral medication	12(75.0%)	14(70.0%)	
Insulin injection	2(12.5%)	1(5.0%)	
BMI (kg/m^2^)-pre op BMI (kg/m^2^)-1 year	23.85 ± 4.07 23.14 ± 4.08	24.36 ± 2.62 23.21 ± 2.02	0.653 0.948
BMI change rate (%)	7.60 ± 10.83	10.99 ± 6.63	0.256
Fasting glucose (mg/dL)—pre op	131.38 ± 62.47	119.35 ± 36.25	0.472
C-peptide (ng/dL)—fast–pre op	2.14 ± 0.95	2.37 ± 1.81	0.644
C-peptide (ng/dL)—fast–1 year	1.69 ± 0.80	1.75 ± 1.40	0.915
Insulin (mIU/L)—fast–pre op	9.74 ± 2.78	11.85 ± 7.01	0.266
HbA1C (%)—pre op	7.63 ± 1.43	6.76 ± 0.87	0.045
HbA1C (%)—post 1 year	7.25 ± 1.17	5.75 ± 0.27	0.002
HOMA_IR—pre op	3.28 ± 1.76	3.64 ± 2.92	0.682
Matsuda index—pre op	4.48 ± 2.32	4.04 ± 3.79	0.696
Insulinogenic index—pre op	0.13 ± 0.14	0.31 ± 0.32	0.038
Disposition index—pre op	0.50 ± 0.42	0.95 ± 0.83	0.057

*T2DM* Type 2 Diabetes Mellitus, *BMI* Body mass index, *HOMA-IR* Homeostatic model assessment of insulin resistance.

Of the 12 patients who underwent total gastrectomy, 5 patients showed T2DM remission, of whom 3 showed remission status and 2 showed partial remission status. In the subtotal gastrectomy group, the proportion of patients with T2DM remission was high, but the difference was not statistically significant (20/36 (55.6%) vs. 5/12 (41.7%): *p* = 0.404).

Table 3 shows the preoperative factors associated with T2DM remission according to logistic regression. In the univariable analysis, there was no predictor associated with T2DM remission. After multivariable model selection, the preoperative insulinogenic index was the only significant predictor of T2DM remission(Odds ratio 133.831(0.905–19,791.308), *p* = 0.045).

**Table 3:** Factors associated with T2DM remission^*^.

	Univariable		Multivariable	
	Odds ratio (95% CI)	*p* value	Odds ratio (95% CI)	*p* value
Age (years, mean ± sd)	0.960 (0.871–1.057)	0.365		
Sex (reference: male)	2.897 (0.290–28.912)	0.365		
BMI (kg/m^2^)	1.192 (0.867–1.640)	0.279		
Gastrectomy type(reference: subtotal)	0.587 (0.095–3.621)	0.566		
C-peptide—pre op	0.966 (0.452–2.063)	0.929		
HOMA-IR—pre op	1.114 (0.762–1.630)	0.577		
Matsuda index—pre op	0.898 (0.685–1.178)	0.437		
Insulogenic index—pre op	44.040 (0.000–4,484,018.395)	0.520	133.831 (0.905–19,791.308)	0.045
Disposition index—pre op	1.599 (0.027–95.561)	0.822		

*Logistic regression model with backward selection.

*BMI* Body mass index, *HOMA-IR* Homeostatic model assessment of insulin resistance.

## Discussion

T2DM is caused by an increase in insulin resistance. Insulin secretory function compensates for the increase in insulin resistance. However, if this compensation does not suffice, the glucose level becomes uncontrollable. If this hyperglycemic stress persists for a long time, pancreatic β cell function is eventually destroyed, resulting in a decrease in insulin secretion. Because Asian people have fewer pancreatic β cells, insulin secretion decreases more severely than in Western people.

The mechanism of gastric cancer surgery as a metabolic surgery has some similarity to that of bariatric RYGP. Remnant stomach volume reduction through gastric resection results in caloric restriction and weight loss. In the case of Billroth II (BII) or Roux-en-Y gastrectomy, food material bypasses the duodenum and proximal jejunum due to the bypassing of the alimentary tract and reaches the distal ileum earlier than is the case in normal individuals. The difference is that gastric cancer patients are relatively less obese; therefore, the effect of weight loss is small compared to that in bariatric patients. In the case of subtotal gastrectomy, the fundus cannot be excised, which is disadvantageous in reducing caloric restriction and ghrelin secretion. The bypass length is shorter than that in bariatric surgery, therefore the effect of enteric hormonal changes may be weaker than that of bariatric RYGP.

The T2DM remission rate after conventional subtotal gastrectomy was reported to be 11.2–22.2%, and after Roux-en-Y reconstruction, it was reported to be 20–30.7%^15,27,34–36^. After LRYG, the remission rate is reported to be 11.6–78.6%^24,25^. In this study, the postoperative remission rate was approximately 47%, and when partial remission was included in this rate, it was approximately 55.6% 1 year after surgery. This remission rate is comparable to the T2DM remission rate in patients with class II obesity^4,5,37^. One of the reasons DM improves after bariatric RYGP is weight loss, which has the effect of improving insulin resistance. Another reason is the effect of changes in hormones at the insuloenteric axis, caused by the change in alimentary tract passing, which is contributes more than weight loss. The foregut hypothesis posits that patients undergoing duodenal bypass experience antidiabetic effects due to decreased anti-incretin factor. The hindgut hypothesis posits that early contact of food material with the distal ileum improves diabetes through the increased and early release of hormones such as glucagon-like peptide-1 (GLP-1) and peptide YY (PYY)^17–21^.

The improvement in T2DM after conventional gastrectomy has been thought to be caused by improved insulin resistance due to weight loss. However, in several previous bariatric studies or gastrectomy studies, there have been reports that pancreatic β cell function plays a more important role in T2DM remission^38–40^. In our study, the preoperative insulinogenic index, which represents insulin secretory function, was the only significant factor influencing T2DM remission. Insulin resistance showed a rapid improvement without weight loss within 1 week after surgery, after which it worsened. Insulin resistance and weight reduction did not show differences 1 year after surgery between the two groups. In contrast, insulin secretion showed a significant difference between both groups 1 year after surgery. In addition, the shape of OGTT graph of the non-remission group showed a normal one, but the glucose value was abnormal. These results indicated that the improvement in T2DM after LRYG caused by increased insulin secretion due to the effect of metabolic surgery was greater than the improvement caused by weight reduction, which is similar to the findings of studies conducted in obese T2DM patients^21^.

**Figure 4:**
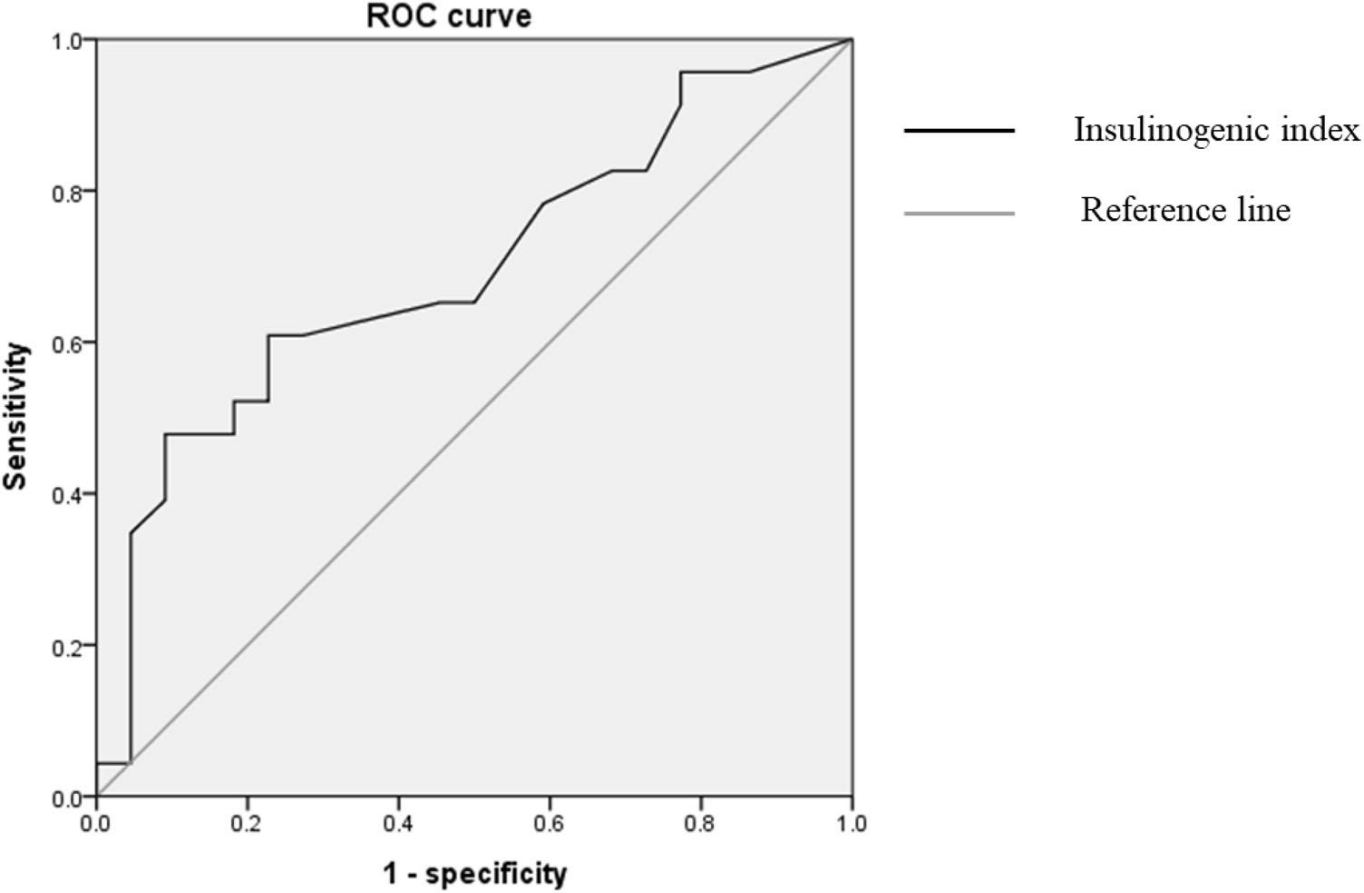
ROC curve of preoperative insulogenic index.

A statistically significant factor related to T2DM remission was the preoperative insulinogenic index. The preoperative insulinogenic index was a useful index (AUC = 0.694), and the insulinogenic index cutoff value was 0.105 (sensitivity = 0.609, specificity = 0.773). (Fig. 4) The normal value of the insulinogenic index is known to be 0.4 or higher; however, LRYG may be considered in patients with an insulinogenic index of 0.1 or higher.

The OGTT test is a very uncomfortable examination because it causes dizziness, nausea, vomiting, etc. It requires several blood samples at one visit, so patient compliance is poor. In this study, various diabetes indices were obtained through OGTT and serum insulin level tests. It is worthwhile to determine which factor is more important in the indication of T2DM remission by calculating the indices. The mixed meal tolerance test cannot calculate various DM indices. Although OGTT is an inconvenient test, it was used for the purpose of this study. All patients were given informed consent for oncometabolic surgery and OGTT test. This study was started in 2010. At the time, it was thought that a biliopancreatic limb and alimentary limb of about 80 cm would be sufficient^25^. Nowadays, longer limbs are accepted as the usual method^41^. If it is performed with a longer biliopancreatic limb and alimentary limb, better results can be expected.

This study was a retrospective study, so the influence of selection bias cannot be excluded. There were some patients who missed follow-up visits, and there was difficulty performing the OGTT test, so some laboratory results were missing. Of the 58 patients, 10 had missing data, and 48 patients were included in the analyses. This study had only 1-year follow-up results and a small number of patients, which limited the statistical power. There is a lack of comparative evaluation of patients’ nutritional status.

The effects of LRYG on weight loss and insulin resistance improvement were not different between the two groups, and the difference in insulin secretory function was the most important factor associated with T2DM remission. The results of this study shows that the effect of LRYG in T2DM patients with low BMI was similar to that of metabolic surgery in obese T2DM patients. Oncometabolic surgery can be one of the optional surgery for T2DM gastric cancer patients with a high possibility of long-term survival and well-preserved pancreatic β cell function, even if they are within normal weight. Besides, these results give us glimpses of the possibility for expanding the indication for metabolic surgery for the T2DM patients with over-weight or normal BMI.
